# The spectrum of thyroid dysfunction in an Australian hepatitis C population treated with combination Interferon-α2β and Ribavirin

**DOI:** 10.1186/1472-6823-5-8

**Published:** 2005-10-12

**Authors:** Huy A Tran, Tracey L Jones, Robert G Batey

**Affiliations:** 1 Hunter Area Pathology Service, John Hunter Hospital, Locked Bag Number 1, Hunter Mail Region Centre, Newcastle, New South Wales 2310, Australia; 2 Hepatitis C Service, Gastroenterology Department, John Hunter Hospital, Locked Bag Number 1, Hunter Mail Region Centre, Newcastle, New South Wales 2310, Australia; 3 Drug And Alcohol Unit, Hunter Area Health Service, John Hunter Hospital, Locked Bag Number 1, Hunter Mail Region Centre, Newcastle, New South Wales 2310, Australia

## Abstract

**Background:**

The study aims to assess the pattern of thyroid response to combination Interferon-α2β (IFN-α) and Ribavirin (RBV) anti-viral therapy in an Australian hepatitis C cohort. These include the prevalence of thyroid dysfunction (TD) including hyperthyroidism and hypothyroidism and their possible predictors, the common overall pattern of thyroid function tests whilst receiving therapy and TD outcomes, and the correlation with HCV status outcome.

**Methods:**

A retrospective analysis of all medical records was performed to assess thyroid function in Hepatitis C Virus (HCV) patients who were treated at the Hunter Area hepatitis C treatment center between 1995 and March 2004. The centre is part of the John Hunter hospital, a major tertiary referral centre in New South Wales, Australia.

**Results:**

There were 272 cases available for review. The prevalence of TD is 6.7 percent and is made up predominantly of females (80 percent). There were 3 (1.1 percent) cases of hyperthyroidism with 2 (67 percent) females. Thirteen out of fifteen (80 percent) cases of hypothyroidism were females with the overall prevalence of 5.5 percent. The majority of hypothyroid patients still required Thyroxine supplement at the end of follow up.

**Conclusion:**

Ninety three percent of HCV treated patients have intact thyroid function at the end of treatment. The predominant TD is hypothyroidism. The predominant pattern of thyrotoxicosis (TTX) is that of thyroiditis although the number is small. Graves' like disease was not observed. People with pre-existing thyroid auto-antibodies should be closely monitored for thyroid dysfunction, particularly hypothyroidism.

## Background

Hepatitis C infection is one of the major epidemics afflicting young adults with more than 150,000 known infected cases in Australia and a notification rate of ~16,000 cases per year in 2002 [1]. In the United States, HCV remains the most common chronic blood-born infection [2,3]. The effective management of the new cases is critically important because, without treatment, approximately 14,000 will develop chronic HCV infection, 6,500 will develop HCV-related cirrhosis, 175 liver failure and 50 with hepatocellular carcinoma. The treatment typically involves the combination of IFN-α and RBV therapy. This is an effective therapy with a 'cure' rate of up to 70% depending on genotype as judged by the negative HCV Ribonucleic Acid (RNA) polymerase chain reaction (PCR) detection [4]. However, no treatment is free from complication and the use of IFN-α is well documented to be associated with TD, the commonest autoimmune disorder associated with IFN-α therapy. This study looks at the combined effect of IFN-α and RBV in an exclusively Australian group of patients with hepatitis C to determine the pattern of thyroid behaviour with direct regards to *eu-, hyper- *and *hypo*-thyroidism. The prevalence of hypothyroidism prevalence and outcome of treatment, in terms of HCV RNA clearance, in direct relationship to thyroid condition are both determined.

## Methods

### Patients

The cases of 272 patients who received combination therapy between 1995 and end of March 2004 at the John Hunter hospital Hepatitis C service were reviewed. All other causes of chronic hepatitis were excluded. No patient had dual Hepatitis B and C. Baseline characteristics of all studied subjects are included in Table 1. Family history of thyroid disease is not available other than in patients who subsequently developed TD.

**Table 1:** Baseline characteristics of 272 patients who received combination IFN-α and RBV therapy for HCV

**Demographics**	
Mean age (years)	42 ± 8
Males	150 (55%)
Caucasians	204 (75%)
Asians	22 (8%)
Weight (kg)	79 ± 18
**HCV Genotype**	

1	136 (50%)
2	22 (8%)
3	103 (38%)
4	11 (4%)
**Liver Function Tests (RR)**	

Albumin (36–48 g/L)	41 ± 2
Serum Bilirubin (2–20 μmol/L)	15 ± 6
Alanine Aminotransferase (< 45 U/L)	133 ± 58
γ-Glutamyl Transpeptidase (1–30 U/L)	98 ± 46
Prothrombin time (11–18 seconds)	15 ± 3
**Haematological Parameters (RR)**	

Haemoglobin (115–165 g/L)	142 ± 16
White cell counts (4.0–11.0 × 10^6^/mL)	7.1 ± 2.0
Platelets (150–400 × 10^9^/mL)	168 ± 49

### Laboratory assays

Serum autoantibodies to anti-thyroglobulin (anti-Tg) and anti-thyroperoxidase (anti-TPO) were measured by agglutination (Serodia-ATG and Serodia-AMC, Fujirebio, Inc., Tokyo, Japan). Titre of less than 1:400 was considered normal for both. Thyroid Stimulating Immunoglobulin (TSI) was measured using cell culture and radio-immunoassay. This is an in-house bioassay using Chinese Hamster Ovary (CHO) cells in culture to detect the presence of thyroid stimulating activity. The CHO cells are transfected with the TSH receptor genes and thus are responsive to TSI. Thyroid-stimulating activity is measured by evaluating the intracellular release of cyclic Adenosine Mono-Phosphate induced by the patient's serum immunoglobulin on the CHO cells. The results are reported as units/mL (U/mL). TSI should be absent in the normal population. A TSI level of <10 is considered negative, 10–50 as weakly, 50–100 as moderate and >100 U/mL as strongly positive.

Third generation serum thyrotropin (TSH), serum free tetra- and free tri-iodothyronine (fT4 and fT3) were determined by two-site sandwich immunoassay using an automated chemiluminescent system (Diagnostic Products Corporation, Immulite 2000). The reference range (RR) for TSH was 0.4–4.0 mU/L, fT4 10.0–26.0 and fT3 3.5–5.5 pmol/L. The coefficients of variations (CV) were 5.0 % and 5.1 % at TSH concentrations of 4.0 mU/L and 10.0 mU/L respectively. For fT4, the CV was 6.5% at 10.0 pmol/L and fT3 8.9% at 3.5 pmol/L.

### Therapy

All patients were treated with combination IFN-α and RBV therapy. The duration of treatment depends on the HCV genotypes; genotypes 2 and 3 were treated for 24 weeks and types 1 and 4 for 48 weeks respectively. Treatment was continued to the end for the latter irrespective of the HCV RNA status at 24 weeks. The dosage for IFN-α was 3 MIU thrice a week with RBV dose ranging from 1000 to 1200 mg daily according to bodyweight.

### Thyroid function assessments

All patients received routine thyroid function tests (TFT) at the start of treatment and at monthly intervals. If there was any concern, then the frequency was increased as clinically indicated. Most, particularly the thyrotoxic group, but not all patients were referred to the Endocrine service for review. Thyroxine supplement was prescribed for hypothyroidism when deemed clinically appropriate. All patients were followed up for a period of 12 months after the completion of anti-viral therapy. Some were followed additionally for a longer period in the Endocrine outpatient service. Thyroid autoantibodies were not routinely performed until the patient developed hypo- or hyperthyroidism on clinical and/or biochemical grounds.

TTX was defined as having TSH of < 0.1 mU/L, fT4 levels > 26.0 and/or fT3 levels > 5.5 pmol/L respectively.

Hypothyroidism, including subclinical hypothyroidism, was defined as having TSH levels > 4.0 mIU/L. Thyroxine supplement was, however, given when deemed clinically appropriate regardless of the degree of TSH elevation.

### Thyroid dysfunction

Thyroid dysfunction (TD) was defined as having *hypo*- or *hyper*-thyroidism, (clinically and/or biochemically based)

### Statistics

Data are presented as percentage and mean ± Standard Error of Mean (SEM).

## Results

### Incidence of thyroid dysfunction in HCV treated group

The majority of patients (93%) had no TD at the end of treatment. Amongst the 272 patients, there were a total of 18 (6.7%) cases of TD: 3 *hyper*-thyroidism and 15 *hypo*-thyroidism.

There were 3 cases of pre-existing hypothyroidism detected at baseline and thus were excluded from the study. All cases were detected at the initial assessment for the treatment of hepatitis C and were shown to have auto-immune hypothyroidism requiring thyroxine supplement. This gave a pre-treatment hypothyroidism prevalence of 1.1% (3/275). Hypothyroidism was seen in 15 (5.5%) with 12 (80%) females after treatment. Thirteen (87%) patients required thyroxine therapy.

TTX was observed in 3 (1.1%) patients whose characteristics are listed in Table 2. Two (67%) were females and all required endocrinology attention. None had any thyroid imaging studies and thus the diagnosis was made on clinical grounds, auto-antibody detection and disease behaviour.

**Table 2:** The pattern of TTX in patients receiving combination IFN-α and RBVNo thyroid nuclear or ultrasonic imaging was available for *all *thyrotoxic cases

**Subjects**	**1**	**2**	**3**
**Gender**	F	F	M
**Age**	26	35	36
**HCV Genotype**	1a	3a	1g
**Regimen duration (weeks)**	48	24	24
**Adverse reactions**	Myalgia, lethargy, emotional lability	None	Insomnia, rash
**Baseline ALT**	184	142	104
**ALT at time of thyroid disease**	33	28	17
**Duration of therapy prior to thyroid disease (weeks)**	16	8	32
**Symptoms of thyroid disease**	Anxiety and depression	Non-specific	Non-specific
**Signs**	No goitre	No goitre	No goitre
**Peak fT4 (pmol/L)**	56.7	57.0	27.0
**Peak fT3 (pmol/L)**	15.1	15.3	12.8
**Thyroid autoantibody status**	• Anti-Tg <1 • Anti-TPO 1:409,600 • TSI <1	• Anti-Tg <1 • Anti-TPO 1:409,600 • TSI <1	• Anti-Tg <1 • Anti-TPO <1:100 • TSI <1
**Thyroid outcomes**	Hypothyroidism on replacement therapy	Hypothyroidism on replacement therapy	Thyroid condition resolved with IFN-α dose reduction
**Hepatic outcomes**	PCR negative	PCR negative	PCR negative

### Thyroid response to combination Interferon-α2B and RBV therapy

#### 1.1 Euthyroid patients

**Figure 1:**
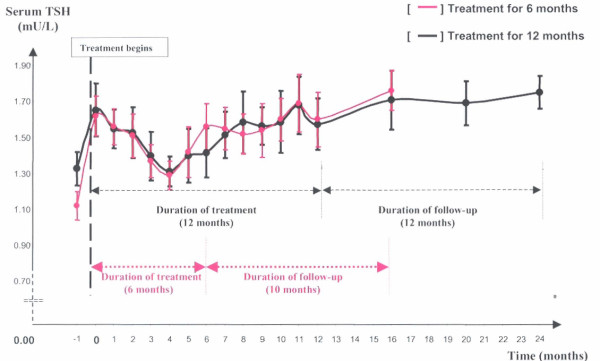
The normal patterns of TSH response in hepatitis C patients during and after receiving combination IFN-α and RBV therapy in the two treated groups at different durations.

Patients who were treated with IFN-α and had normal TFTs were divided into 2 groups according to duration of treatment: at 24 (n = 117) and 48 (n = 136) weeks respectively. Both demonstrated a drop in TSH levels at about 4 months after entry into therapy. Interestingly, none of these patients lowered their TSH level below 0.4 U/L. This was followed by a return to pre-treatment levels and remained so in the follow-up period. Results are illustrated in Table 3 and Figure 1.

**Table 3:** TSH data for the 6 and 12 month treated cohorts who did not develop TD

**Time (months)**	**0**	**2**	**4**	**6**	**16**
**Mean TSH ± SEM (mU/L) for the *6 month treated cohort *n = 117**	**1.62 ± 0.11**	**1.51 ± 0.12**	**1.29 ± 0.08**	**1.56 ± 0.13**	**1.76 ± 0.11**
	**0**	**4**	**8**	**12**	**16**
**Mean TSH ± SEM (mU/L) for the *12 month treated cohort n = 136***	**1.65 ± 0.15**	**1.31 ± 0.08**	**1.58 ± 0.17**	**1.57 ± 0.14**	**1.71 ± 0.16**

#### 1.2 Hypothyroid patients

All hypothyroid patients had non-specific symptoms and relevant data are listed in table 4. Free T3 levels were not routinely measured in the presence of hypothyroidism. Anti-TPO antibodies were detected in 6 out of 15 hypothyroid patients (40%) with titres ranging from 1: 6,400 to 1: 409,600. The rest had titres <1:400. Baseline autoantibodies were not routinely requested at the initial assessment.

**Table 4:** Data for hypothyroid patients receiving combination therapy for Hepatitis C infection

**Number of subjects**	15 *(9 treated for 48 weeks and 6 for 24 weeks)*
**Gender**	3 M : 12 F
**Mean age**	44 ± 2
**Positive family history of thyroid disease (in first degree relatives)**	8/18 (44%)
**Duration of IFN-α before thyroid disease developed (months)**	4.8 ± 1.2
**Mean TSH levels (mU/L)**	29.7 ± 8.8
**Mean fT4 levels (pmol/L)**	3.5 ± 0.9
**The presence of TPO-autoantibodies**	6/15 (40%)
**Thyroxine requirement at the 12-month follow-up**	13/15 (87%)
**PCR negativity at the end of treatment**	15/15 (100%)

#### 1.3 Hyperthyroid patients

All 3 thyrotoxic patients had suppressed TSH levels with varying levels of fT4 and fT3. The time to development of TTX was variable but most occurred whilst receiving treatment. Anti-TPO antibodies were elevated in patients 1 and 2 (2 out of 3 cases) and both behaved in a thyroiditis fashion. Hypothyroidism developed rapidly and Thyroxine supplement was subsequently required within 1 month. In patient 3, despite the negative antibody results, the TTX responded to a reduction in IFN-α dosage suggesting an IFN-α/autoimmune mediated mechanism. TSI was undetectable in all 3 patients.

All (100%) patients with thyroid dysfunction cleared their hepatitis C infection based on the aforementioned criteria against a background overall response rate of ~75% to the combination anti-viral therapy in our cohort.

## Discussion

This is the first report to characterise thyroid responses to combination anti-viral treatment in an Australian cohort with hepatitis C infection. The prevalence of TD is ~7% of which 83% is hypothyroidism. The predominant gender is female at 80%. This is consistent with previous reports relevant to this condition [5-7]. The prevalence range is broad reflecting the differences in the definition of TD, some are biochemically based, others clinically based or both.

Overall, 93% (254/272 cases) of patients have normal thyroid outcome. In the majority of cases, the TSH levels remain within reference range throughout. Levels decrease slightly at 4 months but return to pre-existing level with the continuation of treatment irrespective of the duration of therapy. The range of TSH values is narrow ranging between 1.15 at the 4-month nadir and 2.03 at 16 months in 95% of cases whilst undergoing therapy. FT4 levels were not routinely measured in the presence of normal TSH levels which remain the single best assessment of thyroid function in the presence of intact hypothalamo-pituitary-thyroid axis [8], expected in this sub-group. Whilst it is tempting to attribute the decrease in TSH level during treatment to IFN-α and RBV, it is likely that non-thyroidal illness is the main underlying pathogenesis. Other co-morbidities associated with combination treatment (but particularly IFN-α) include fever, headache, depression and neuropsychiatric disturbance etc... all contribute to the changes in TFTs [9].

In TTX, the number is too small to arrive at any definitive conclusions. Only the destructive thyroiditis form is observed in this study. Goitre is absent in all cases. In patients 1 and 2, this type of hyperthyroidism rapidly converts to hypothyroidism in the presence of high auto-antibody titre and subsequently required permanent Thyroxine supplement. In patient 3, the TTX was mild and resolved with IFN dose reduction. This was presumed to be IFN-α/autoimmune mediated despite the absence of auto-antibodies. This pattern has been observed elsewhere [10-12]. Unfortunately, radioactive iodine uptake scans were not performed so that the diagnoses could be unequivocally confirmed. TTX resembling Graves' disease was not observed. In this scenario, TTX is uncommon and thus each case should be dealt with on its own merits. The index of clinical suspicion should remain high whilst on therapy. Management is best done in a specialist Endocrine clinic, with destructive thyroiditis being more common, often resulting in permanent hypothyroidism.

Hypothyroidism is clearly the commoner cause of TD in combination therapy. It has been suggested that the virus itself may play a role in the development of hypothyroidism in IFN-naïve patients as the prevalence is higher than the general population [13]. Also, if IFN-α alone is thought to cause hypothyroidism, the prevalence of hypothyroidism should be higher than in IFN-naïve patients and this is the case of this report with a 5 folds increase after treatment. However, this observation has not been consistent in the general literature, see Table 5[14-19]. To further add to the complexity of the situation, hypothyroidism is also more frequent in patients having combination therapy of IFN-α and RBV [20] (as opposed to IFN-α treated alone). Whilst HCV is well known to induce a higher prevalence of auto-antibody, this does not necessarily translate into hypothyroidism (either clinical or subclinical) [14]. It would have been additionally interesting to characterize, in parallel, the pattern of thyroid function in our HCV patients who did not receive HCV treatment for direct comparison. Unfortunately these data are not available.

**Table 5:** The prevalence of hypothyroidism in the IFN-naïve and IFN-treated HCV patients compared with the general populations. The prevalence of elevated TSH levels in the various population groups include: Australia ~2.0% [17], the United States of America ~7.3% [18], Spain ~2.0% [18], Japan ~1.3% [18], Great Britain ~7.3% [18] and Italy ~1.7% [19]

**Studies N = number of subjects**	**Prevalence of hypothyroidism (before IFN)**	**Prevalence of hypothyroidism (after IFN)**
Marcellin et al. [5] N = 74	0	7.2
Preziati et al. [15] N = 78	0	22.8
Imigawa et al. [14] N = 58	0	3.4
Marazuela et al. [7] N = 207	4.7	2.8
**This work N = 272**	**1.1%**	**7.5%**
Baudin et al. [16] N = 68	0	7.3
Antonelli et al. [13] N = 630	13%	N/A

The pathogenesis remains poorly understood but IFN-α is thought to be related to have a direct inhibitory effect on thyrocytes preventing hormonogenesis and secretion. Another postulate is immunostimulation in the presence of hepatitis C infection. This is thought to include activation of lymphocytes and natural killer cells, increased production of Tumor Necrosis Factor, IFN-α, Interleukin and other cytokines and increased production of immunoglobulins [21]. All lead to the development of thyroid auto-antibodies with complete destruction and consequently permanent hypothyroidism in genetically susceptible individuals. Although this does occur in hepatitis B IFN-α treated patients, the prevalence of hypothyroidism is much lower. This suggests that the hepatitis C virus or its genome plays an integral part the development of thyroid dysfunction [10]. The virus itself has been postulated to induce thyroid auto-antibodies by ways of generating high *endogenous *IFN levels triggering off autoimmune thyroid disease in susceptible individuals, similar to Coxsackievirus. This virus and others have been shown to induce a higher level of endogenous IFN-α levels which have been associated with other auto-immune diseases such as type 1 diabetes [22]. When IFN-α is administered exogenously, another layer of complexity is added. It is possible but purely speculative that exogenous IFN-α synergises with the endogenous source, thus exaggerating the effect on the thyroid thus causing additional hypothyroidism.

Whatever the underlying pathophysiological process is, the major factors contributing to hypothyroidism includes the female gender with a relative risk ranging between 3–7 times [23] and the presence of anti-TPO antibodies (Ab). These factors feature prominently in our report although the prevalence of anti-TPO Ab is lower. The time to onset of biochemical hypothyroidism means at about 5 months, similar to other cohorts [6].

When IFN-α triggers destructive thyroid disease in genetically susceptible individuals, some may recover but the majority does not in our study. Thyroxine requirement therefore is expected to be long term as illustrated in 87% (13/15) of our cases. All 6 (100%) patients where anti-TPO antibodies are detected, are on thyroxine at the completion of follow up. The symptomatology of hypothyroidism is non-specific and often overlaps with IFN-α side-effects. The diagnosis often requires biochemical testing with some patients receiving thyroxine supplement at mildly elevated concentrations of TSH and/or low/normal fT4 levels. Where thyroxine was deemed necessary and thus given, all were continued with thyroxine supplement at 12-month follow-ups suggesting that thyroid damage is permanent although longer follow-up is preferred.

All patients with TD cleared their HCV infection up until end of follow-up. The number is too small to make any conclusion about the advantage of having thyroid dysfunction when it comes to eradicating the virus. Hypothetically, these patients may have developed an exaggerated response which helps to clear the virus and in the process induces thyroid dysfunction, an unfortunate but acceptable side-effect of the treatment modality. Whilst this is highly plausible, it has not been supported by other investigators [24].

## Conclusion

IFN-α in combination with RBV therapy, by and large, does not cause thyroid dysfunction in the majority of HCV patients undergoing such treatment. Patients undergoing such therapy can expect a small decrease in TSH levels, which remained in the RR however, without going into frank hyper- or hypothyroidism. The incidence of TD in HCV patients receiving combination anti-viral therapy is generally small with hypothyroidism being the most common. All patients should be assessed thoroughly for relevant risk factors and monitoring for dysfunction at the appropriate time frame. Hyperthyroidism is uncommon and thus is best managed in a specialised Endocrine clinic. The important points derived from this study are summarized in Table 6.

**Table 6:** Major summary points from the study

1. The majority of patients with HCV undergoing combination IFN-α and RBV therapy have normal thyroid function.
2. The commonest cause of thyroid dysfunction is hypothyroidism, a ratio of 4:1 compared with hyperthyroidism.
3. The average time from the start of anti-viral treatment to hypothyroidism is ~4–5 months, suggesting that this is the critical time to carry out thyroid testing.
4. Predisposing risk factors include female gender, family history of thyroid disease and existing thyroid auto-antibodies, especially anti-TPO antibodies. In this situation, initial TSH should be performed to exclude pre-existing hypothyroidism.
5. Hyperthyroidism is less common and each should be managed on its own merits in a specialised Endocrine service.

## Competing interests

The author(s) declare that they have no competing interests.

## Authors' contributions

TLJ gathered, provided the data and participated in the discussion and drafting of the manuscript. RGB participated in the scientific discussion and drafting on the manuscript. HAT conceived the study, participated in its design, assisted with data collection, coordinated and helped to draft the manuscript. All authors read and approved the revised manuscript.

## Pre-publication history

The pre-publication history for this paper can be accessed here:


